# A Statistical Method for Synthesizing Meta-Analyses

**DOI:** 10.1155/2013/732989

**Published:** 2013-10-01

**Authors:** Liansheng Larry Tang, Michael Caudy, Faye Taxman

**Affiliations:** ^1^Department of Statistics, George Mason University, Fairfax, VA 22030, USA; ^2^Department of Criminology, Law and Society, George Mason University, Fairfax, VA 22030, USA

## Abstract

Multiple meta-analyses may use similar search criteria and focus on the same topic of interest, but they may yield different or sometimes discordant results. The lack of statistical methods for synthesizing these findings makes it challenging to properly interpret the results from multiple meta-analyses, especially when their results are conflicting. In this paper, we first introduce a method to synthesize the meta-analytic results when multiple meta-analyses use the same type of summary effect estimates. When meta-analyses use different types of effect sizes, the meta-analysis results cannot be directly combined. We propose a two-step frequentist procedure to first convert the effect size estimates to the same metric and then summarize them with a weighted mean estimate. Our proposed method offers several advantages over existing methods by Hemming et al. (2012). First, different types of summary effect sizes are considered. Second, our method provides the same overall effect size as conducting a meta-analysis on all individual studies from multiple meta-analyses. We illustrate the application of the proposed methods in two examples and discuss their implications for the field of meta-analysis.

## 1. Introduction

The last two decades have seen an exponential growth in the popularity of meta-analyses across scientific disciplines including medical research [1] and diagnostic medicine [2]. The purpose of meta-analysis is to assess the consistency and robustness of findings across populations, settings, and contextual factors in order to help ensure that a practice is likely to produce similar results when it is implemented. A single study cannot determine, with certainty, that an intervention works or does not work. Instead, studies that are combined together, across different settings, and conducted over time can establish a pattern of consistent findings that may be useful to justify new or refined practice. Several studies combined can establish both significance and repeatability of results [3]. Common statistical methods to combine studies in meta-analyses are based on a fixed-effects model which assumes a homogeneous treatment effect among studies or a more general random-effects model which allows heterogeneity among studies [4].

Multiple meta-analyses are sometimes conducted to investigate the effect of the same topic or intervention. The synthesis of these meta-analyses can be used to highlight what is known on a particular topic or intervention or what can contribute to more complete understanding of the extant empirical evidence [5]. However, the summarizing of meta-analyses can be very challenging because existing meta-analyses may be conflicting, may be reported differently or incompletely across studies, or may be more or less valid based on the quality of the research synthesis methods that were employed in conducting the reviews. This raises challenges for interpreting and drawing conclusions about what the results of these studies mean and how they should be used to inform future research, theory development, and practice. Systematic reviews are further complicated when a diverse array of health-relevant outcomes are assessed. This is essentially the same issue researchers and practitioners face when attempting to summarize primary studies, the same issue that makes meta-analysis appealing.

Several nonstatistical approaches to summarizing meta-analyses of the same topic or intervention are currently available. The Cochrane Collaboration has developed a set of recommended procedures for conducting overviews of reviews when multiple meta-analyses exist regarding different treatments for the same clinical condition [6–8]. Nonstatistical approaches to summarizing multiple reviews, which may be used for meta-analyses, include vote counting and using decision algorithms to identify the review(s) that are most salient [7]. However, narrative reviews can be far too subjective to reflect the knowledge that has been gained through research [9]. An ideal statistical approach to summarizing meta-analyses is to conduct a new meta-analysis of all primary studies included in multiple meta-analyses. That is, individual studies in all the related meta-analyses are identified, and their effect sizes are combined to calculate a summary statistic using meta-analytic techniques such as the random-effects model in DerSimonian and Laird [4]. However, while this approach is ideal and appealing when there are only two or three meta-analyses to combine, doing so is not as efficient as directly summarizing effect sizes reported in existing meta-analyses on similar topics. Recently, Hemming et al. [10] provided a Bayesian method to summarize multiple reviews. Their method assumes that estimated summary effect sizes follow a random-effects model based on exchangeability assumption. Differing types of effect sizes are not considered in their method. In addition, the authors did not study the fixed-effects models which may lead to nice properties for the combined overall effect size.

In this paper we start with describing methods to meta-analyze effect size estimates from individual studies extracted from several existing meta-analyses. However, the methods require a substantial amount of time and resources. Thus, we also introduce a more efficient statistical method to directly summarize information from existing meta-analyses without going back to individual studies. The paper is organized as follows. In Section 2 we introduce the notations and our method for synthesizing meta-analyses with the same type of summary statistics. We also describe two-step methods for combining different types of summary statistics commonly used in meta-analyses. We start with the fixed-effects model and show that summarizing the reviews yields the same overall effect size as conducting a meta-analysis on all possible individual studies. We then describe the method for the random-effects model.  Section 3 applies the proposed method to two examples.  Section 4 discusses some potential applications of this method.

## 2. Methods

 The use of quantitative research synthesis techniques such as meta-analysis overcomes many of the limitations of traditional narrative literature reviews. Meta-analysis provides an objective and quantitative approach to research synthesis by taking into account factors such as sample size, magnitude, and direction of relationships and the methodological quality of the various studies analyzed [11–13]. Meta-analyses are not limited by a reliance on traditional indicators of statistical significance but instead rely upon effect sizes to give a picture of the size and scope of the impact of an intervention [14]. However, the more meta-analyses that are conducted on a given topic, the more difficult it can be to analyze and determine the cumulative findings from a body of literature.

Calculating the summary effect sizes across existing meta-analyses is a potentially valuable way to synthesize the knowledge base on a given topic. This process should be guided by the same rule that guides traditional meta-analysis which requires that studies being combined should be assessing a common clinical research question. Suppose several meta-analyses consider a common clinical question, and use similar search criteria to include and exclude individual studies. Summarizing these meta-analyses can provide better understanding of the clinical question than any single meta-analysis.

When multiple meta-analyses address the same topic, we propose that researchers should explore methods to synthesize the summary effect sizes from the existing meta-analyses. This proposed approach only requires summary effect sizes and their variances which are reported in the existing meta-analyses. The calculated overall effect size for the combined meta-analyses is essentially a weighted average of the summary effect sizes with the weights being the inverse of the variances; this approach is analogous to the techniques used to conduct a meta-analysis of primary studies.

Popular effect size estimates used in meta-analyses include standardized mean differences, odds ratios, and correlation coefficients [14]. In this section, we first consider the simplest case of combining multiple meta-analyses when all analyses use the same type of effect size estimates in a fixed-effects model. We then extend the method to the more commonly used random-effects model. Finally, we present a method for combining meta-analyses with different types of summary statistics in the fixed-effects or random-effects model.

### 2.1. Combining Effect Sizes from the Same Measure

#### 2.1.1. Synthesizing All Individual Studies

Suppose *J* meta-analyses using similar search criteria on a common topic are identified and need to be combined to obtain an overall summary statistic. We use *θ*_*ij*_ and ${\hat{\theta }}_{ij}$ to denote a true effect parameter and its estimate, respectively, for study *i*, *i* = 1,…, *k*_*j*_, in meta-analysis *j*. The estimate ${\hat{\theta }}_{ij}$ is commonly assumed to follow a normal distribution as ${\hat{\theta }}_{ij}∼N(\theta_{ij},\sigma_{ij}^{2})$ where *σ*_*ij*_^2^ is the variance of ${\hat{\theta }}_{ij}$. The variance estimator, ${\hat{\sigma }}_{ij}^{2}$, obtained from the study tends to be smaller for a larger sample size. If all ${\hat{\theta }}_{ij}$'s and ${\hat{\sigma }}_{ij}^{2}$'s are available, researchers can conduct a meta-analysis using existing statistical methods based on a random-effects model (REM) or a fixed-effects model (FEM). As a popular model in meta-analysis, the REM assumes heterogeneity among *θ*_*ij*_ and is given as follows:

(1) $$\begin{array}{c} {\hat{\theta }}_{ij}=\theta_{j}+\epsilon_{ij},\\ \theta_{j}=\theta +u_{j}, \end{array}$$

where *ϵ*_*ij*_ ~ *N*(0, *σ*_*ij*_^2^) and *u*_*j*_ ~ *N*(0, *τ* ^2^). Here *σ*_*ij*_^2^ is estimated using the sample variance ${\hat{\sigma }}_{ij}^{2}$ from study *i* in meta-analysis *j*. Conventionally, the true variance *σ*_*ij*_^2^ is assumed to be known and equal to ${\hat{\sigma }}_{ij}^{2}$, and to simplify the notation we will use *σ*_*ij*_^2^ instead of ${\hat{\sigma }}_{ij}^{2}$ throughout the paper. The random effect, *u*_*j*_, represents heterogeneous *θ*_*j*_ among all studies, and its variance can be estimated using a moment estimator ${\hat{\tau }}^{2}$ by DerSimonian and Laird [4]:

(2) $$\begin{array}{c} {\hat{\tau }}^{2}=\frac{Q-\left(\sum_{j=1}^{J}k_{j}-1\right)}{\sum_{i,j}w_{ij}-\sum_{i,j}w_{ij}^{2}/\sum_{i,j}w_{ij}}, \end{array}$$

where

(3) $$\begin{array}{c} Q=\underset{i,j}{\sum }w_{ij}{\hat{\theta }}_{ij}^{2}-\frac{{\left(\sum_{i,j}w_{ij}{\hat{\theta }}_{ij}\right)}^{2}}{\sum_{i,j}w_{ij}}, \end{array}$$

and the weight *w*_*ij*_ is given by 1/*σ*_*ij*_^2^ indicating a larger weight for a larger sample size. The moment estimate, ${{\hat{\tau }}_{j}}^{2}$, should always be nonnegative. If a negative value of ${\hat{\tau }}_{j}$ is obtained, $\hat{\tau }$ is set to 0 according to DerSimonian and Laird [4]. Using $w_{ij}^{*}=1/(\sigma_{ij}^{2}+{\hat{\tau }}^{2})$ as a weight for study *i* in meta-analysis *j*, an estimate for the summary effect *θ* is given by a weighted average of effect estimates:

(4) $$\begin{array}{c} \hat{\theta }=\frac{\sum_{j=1}^{J}\sum_{i=1}^{k_{j}}w_{ij}^{*}{\hat{\theta }}_{ij}}{\sum_{j=1}^{J}\sum_{i=1}^{k_{j}}w_{ij}^{*}}. \end{array}$$

In the FEM, the random-effect component in (1) is 0 by assuming true effect parameters are homogenous across studies, and the REM in (1) becomes

(5) $$\begin{array}{c} {\hat{\theta }}_{ij}=\theta +\epsilon_{ij}. \end{array}$$

The estimate for *θ* can be obtained using (4) by setting $\hat{\tau }=0$.

#### 2.1.2. Synthesizing Meta-Analyses

 Obtaining $\hat{\theta }$ with results from all individual studies as in (4) is an ideal way to synthesize multiple meta-analyses. In the traditional meta-analysis, hundreds of studies are often available on a similar topic, and when authors of some individual studies are not contactable, criteria can be set accordingly to exclude these studies. However, extracting individual study data requires tremendous time and resources. Alternatively, we propose to directly summarize meta-analyses. We start with showing the equivalence of synthesizing meta-analyses and synthesizing all individual studies under the simple FEM which assumes the same effect parameter, *θ*, across studies. The effect parameter *θ*_*j*_ in meta-analysis *j* is estimated using

(6) $$\begin{array}{c} {\hat{\theta }}_{j}=\frac{\sum_{i=1}^{k_{j}}w_{ij}{\hat{\theta }}_{ij}}{\sum_{i=1}^{k}w_{ij}}, \end{array}$$

where *w*_*ij*_ = 1/*σ*_*ij*_^2^. The variance estimate of ${\hat{\theta }}_{j}$ is given by $\text{var}({\hat{\theta }}_{j})=1/\sum_{i=1}^{k}w_{ij}$. Using $w_{j}=1/\text{var}({\hat{\theta }}_{j})$ as the weight for meta-analysis *j*, we can obtain a weighted average of *J* meta-analysis results as follows:

(7) $$\begin{array}{c} {\hat{\theta }}_{f}=\frac{\sum_{j=1}^{J}w_{j}{\hat{\theta }}_{j}}{\sum_{j=1}^{J}w_{j}}. \end{array}$$

After some math, we see that the expression above is the same as (4) with $\hat{\tau }=0$. The result implies that conducting a meta-analysis by pooling summary effect sizes of the meta-analyses of interest is equivalent to a meta-analysis of combining all individual studies from these meta-analyses. The variance of the weighted average is given by $\text{var}({\hat{\theta }}_{f})=1/(\sum_{j=1}^{J}w_{j})$.

The assumption of the same effect parameter across the studies may not be realistic considering studies' heterogeneous characteristics. This assumption is relaxed in the REM as shown in (1). Using the data from meta-analysis *j*, the parameter *τ* accounting for the random component is estimated by the following moment estimator:

(8) $$\begin{array}{c} {\hat{\tau }}_{j}^{2}=\max\left\{0,\frac{Q_{j}-\left(k_{j}-1\right)}{\sum_{i}w_{ij}-\left(\sum_{i}w_{ij}^{2}/\sum_{i}w_{ij}\right)}\right\}, \end{array}$$

where

(9) $$\begin{array}{c} Q_{j}=\sum_{i}w_{ij}{\hat{\theta }}_{ij}^{2}-\frac{\left(\sum_{i}w_{ij}{\hat{\theta }}_{ij}^{2}\right)}{\sum_{i}w_{ij}}. \end{array}$$

The moment estimator ${\hat{\tau }}_{j}^{2}$ has the same expression as the one given in (8) except that it is now obtained using only data from meta-analysis *j*. Meta-analysis *j* then uses ${\tilde{w}}_{ij}^{*}=1/(\sigma_{ij}^{2}+{\hat{\tau }}_{j}^{2})$ as a weight for study *i* to obtain an estimate for the summary effect *θ*_*j*_ by

(10) $$\begin{array}{c} {\hat{\tilde{\theta }}}_{j}^{*}=\frac{\sum_{i=1}^{k_{j}}{\tilde{w}}_{ij}^{*}{\hat{\theta }}_{ij}}{\sum_{i=1}^{k_{j}}{\tilde{w}}_{ij}^{*}}. \end{array}$$

To synthesize *J* meta-analyses, a possible way is to use ${\tilde{w}}_{j}^{*}=1/\sum_{i=1}^{k_{j}}{\tilde{w}}_{ij}^{*}$ as the weight for meta-analysis *j* and calculate a weighted average of ${\hat{\theta }}_{j}$'s as follows:

(11) $$\begin{array}{c} {\hat{\theta }}_{r}=\frac{\sum_{j=1}^{J}{\tilde{w}}_{j}^{*}{\hat{\tilde{\theta }}}_{j}^{*}}{\sum_{j=1}^{J}{\tilde{w}}_{j}^{*}}. \end{array}$$

Comparison between the estimate above and the one in (4) shows some difference. We can write (4) as the weighted average of meta-analyses summary statistics in the following expression:

(12) $$\begin{array}{c} \hat{\theta }=\frac{\sum_{j=1}^{J}w_{j}^{*}{\hat{\theta }}_{j}^{*}}{\sum_{j=1}^{J}w_{j}^{*}}, \end{array}$$

where

(13) $$\begin{array}{c} {\hat{\theta }}_{j}^{*}=\frac{\sum_{i=1}^{k_{j}}w_{ij}^{*}{\hat{\theta }}_{ij}}{\sum_{i=1}^{k_{j}}w_{ij}^{*}}, \end{array}$$

and *w*_j_* = 1/∑_*i*=1_^*k*_*j*_^ *w*_ij_*. A close look at ${\hat{\tilde{\theta }}}_{j}^{*}$ and ${\hat{\theta }}_{j}^{*}$ reveals a slight difference. This is due to different weight estimates, ${\tilde{w}}_{ij}^{*}$ and *w*_ij_*, where the former uses studies in meta-analysis *j* to estimate *τ* and the latter pools studies from all meta-analyses to estimate *τ*. In practice, a well-planned meta-analysis includes a large number of studies, and ${\hat{\tau }}_{j}^{2}$'s and ${\hat{\tau }}^{2}$ should all be close to the true parameter *τ*. Subsequently, the difference between ${\hat{\theta }}_{r}$ and $\hat{\theta }$ can be possibly ignored. Thus, although the weights used in (11) may not be as accurate as the ones obtained by pooling all individual studies, ${\hat{\theta }}_{r}$ is still a statistically sound estimate for *θ*.

### 2.2. Combining Effect Sizes from Different Types of Statistics

The summary statistics in meta-analyses may be of different types. When individual studies in a meta-analysis have continuous outcomes, the statistic may appear in the form of the sample mean difference. Sometimes, other studies address the same topic, but the outcome is dichotomized, and the odds ratio is commonly used to summarize the difference between two groups. The Pearson correlation coefficient *r* is frequently used to evaluate the correlation between two continuous variables. When one variable is a continuous outcome and the other variable is dichotomized to indicate the group status, *r* can be used to compare outcomes between two groups. When meta-analyses addressing the same topic use different types of statistics, one should convert these statistics to the same type of summary statistics before synthesizing them.

#### 2.2.1. Synthesizing All Individual Studies

We now discuss how to combine meta-analyses with these types of summary statistics using a two-step procedure. The first step is to convert various types of statistics to the same type. In this paper, we discuss how to convert the log-transformed odds ratio (OR) and the correlation coefficient to the same scale of the sample mean difference. The log-transformed OR can be converted to the sample mean difference using their linear relationship as will be discussed. However, a Taylor expansion is needed to convert the correlation coefficient to the sample mean difference via linear relationship. We focus on the linear relations because the weighted average in a meta-analysis is a linear combination of effect estimates, and preserving the linear nature in the conversion will facilitate the future calculation for synthesizing the meta-analyses.

Suppose meta-analysis *j*, *j* = 1,…, *J*, is conducted using the standardized sample mean difference ${\hat{\theta }}_{j,m}$ with its variance of *σ*_*j*,*m*_^2^ and meta-analysis *j*′, *j*′ = 1,…, *J*′, has the estimated log-transformed odds ratio ${\hat{\theta }}_{j^{\prime },\text{OR}}$ as the summary effect and its sample variance of *σ*_*j*′,OR_^2^.

Chinn gives the detailed discussion on how to convert between the standardized mean difference and the estimated odds ratios from individual studies [15]. Since the log-transformed odds ratio, ${\hat{\theta }}_{ij^{\prime },\text{OR}}$, in study *i* of meta-analysis *j* follows approximately a logistic distribution which differs from a standard normal distribution mainly in the tail area, dividing ${\hat{\theta }}_{ij^{\prime },\text{OR}}$ by $\pi /\sqrt{3}$, or 1.81, converts ${\hat{\theta }}_{ij^{\prime },\text{OR}}$ to an approximate standardized sample mean difference.

In meta-analysis *j*′′, *j*′′ = 1,…, *J*′′, with correlation coefficients, the coefficient ${\hat{\theta }}_{ij^{\prime \prime },r}$ from study *i* is converted to Fisher's *z*-score ${\hat{\theta }}_{ij^{\prime \prime },z}$ before the further calculation [14]. The conversion is given by

(14) $$\begin{array}{c} {\hat{\theta }}_{ij^{\prime \prime },z}=\frac{1}{2}\ln\left(\frac{1+{\hat{\theta }}_{ij^{\prime \prime },r}}{1-{\hat{\theta }}_{ij^{\prime \prime },r}}\right). \end{array}$$

Since a correlation coefficient ${\hat{\theta }}_{r}$ can be converted to a standardized mean difference ${\hat{\theta }}_{m}$ using ${\hat{\theta }}_{m}=2{\hat{\theta }}_{r}/\sqrt{1-{\hat{\theta }}_{r}^{2}}$, we can convert Fisher's *z*-score ${\hat{\theta }}_{z}$ to ${\hat{\theta }}_{m}$ using ${\hat{\theta }}_{m}=e^{{\hat{\theta }}_{z}}-e^{-{\hat{\theta }}_{z}}$. The relationship above between ${\hat{\theta }}_{z}$ and ${\hat{\theta }}_{m}$ is nonlinear. The second order Taylor expansion of

(15) $$\begin{array}{c} {\hat{\theta }}_{m}=1+{\hat{\theta }}_{z}+\frac{{\hat{\theta }}_{z}^{2}}{2}-\left(1-{\hat{\theta }}_{z}+\frac{{\hat{\theta }}_{z}^{2}}{2}\right)=2{\hat{\theta }}_{z} \end{array}$$

gives the approximate linear relationship between ${\hat{\theta }}_{z}$ and ${\hat{\theta }}_{m}$.

After the effect estimates are converted to the same scale of ${\hat{\theta }}_{j,m}$, they can be used to estimate the overall effect parameter *θ* in the following REM model:

(16) $$\begin{array}{c} {\hat{\theta }}_{ij,m}=\theta_{ij}+\epsilon_{ij},\;\;\frac{{\hat{\theta }}_{ij^{\prime },\text{OR}}}{1.81}=\theta_{ij^{\prime }}+\epsilon_{ij^{\prime }},\\ 2{\hat{\theta }}_{ij^{\prime \prime },z}=\theta_{ij^{\prime \prime }}+\epsilon_{ij^{\prime \prime }},\;\;\theta_{ij}=\theta +u_{ij},\\ \theta_{ij^{\prime }}=\theta +u_{ij^{\prime }},\;\;\theta_{ij^{\prime \prime }}=\theta +u_{ij^{\prime \prime }}, \end{array}$$

where *ϵ*_*ij*_ ~ *N*(0, *σ*_*ij*_^2^), *ϵ*_*ij*′_ ~ *N*(0, *σ*_*ij*′_^2^), *ϵ*_*ij*′′_ ~ *N*(0, *σ*_*ij*′′_^2^), and *u*_*ij*_, *u*_*ij*′_, *u*_*ij*′′_ ~ *N*(0, *τ* ^2^).

In the second step, ${\hat{\theta }}_{ij,m}$, ${\hat{\theta }}_{ij,\text{OR}}$, and ${\hat{\theta }}_{ij,z}$ are combined to obtain the overall summary effect estimate $\hat{\theta }$. Let *w*_*ij*′_ = 1/*σ*_*ij*_^2^, *w*_*ij*′_/*σ*_*ij*′_^2^, and *w*_*ij*′′_ = 1/*σ*_*ij*′′_^2^. Again, the parameter *τ* ^2^ can be estimated by a moment estimator ${\hat{\tau }}^{2}$ similar to (8) using all effect estimates and their variances:

(17) $$\begin{array}{c} {\hat{\tau }}^{2}=\max\left\{0,\frac{Q-\left(\sum_{j=1}^{J}k_{j}+\sum_{j^{\prime }=1}^{J}k_{j^{\prime }}+\sum_{j^{\prime \prime }=1}^{J}k_{j^{\prime \prime }}-1\right)}{w-w^{2}/w}\right\}, \end{array}$$

where

(18) w=∑i,jwij+∑i,j′wij′+∑i,j′′wij′′,w2=∑i,jwij2+∑i,j′wij′2+∑i,j′′wij′′2,Q=∑i,jwijθ^ij,m2+∑i,j′wij′(θ^ij′,OR1.81)2+∑i,j′′wij′′(2θ^ij′′,r)2 −1w(∑i,jwijθ^ij,m+∑i,j′wij′θ^ij′,OR1.81+∑i,j′′2wij′′θ^ij′′,r)2.

The resulting estimate for the summary effect *θ* using the REM is given by

(19) $$\begin{array}{c} \hat{\theta }=\frac{\sum_{i,j}w_{ij}^{*}{\hat{\theta }}_{ij,m}+\sum_{i,j^{\prime }}w_{ij^{\prime }}^{*}{\hat{\theta }}_{ij^{\prime },\text{OR}}/1.81+\sum_{i,j^{\prime \prime }}2w_{ij^{\prime \prime }}^{*}{\hat{\theta }}_{ij^{\prime \prime },r}}{\sum_{i,j}w_{ij}^{*}+\sum_{i,j^{\prime }}w_{ij^{\prime }}^{*}+\sum_{i,j^{\prime \prime }}w_{ij^{\prime \prime }}^{*}}, \end{array}$$

where $w_{ij^{\prime }}^{*}=1/(\sigma_{ij}^{2}+{\hat{\tau }}^{2})$, $w_{ij^{\prime }}^{*}=1/(\sigma_{ij^{\prime }}^{2}+{\hat{\tau }}^{2})$, and $w_{ij^{\prime \prime }}^{*}=1/(\sigma_{ij^{\prime \prime }}^{2}+{\hat{\tau }}^{2})$. The variance of  $\hat{\theta }$  is given by $\text{var}(\hat{\theta })=1/(\sum_{i,j}w_{ij}^{*}+\sum_{i,j^{\prime }}w_{ij^{\prime }}^{*}+\sum_{i,j^{\prime \prime }}w_{ij^{\prime \prime }}^{*})$.

#### 2.2.2. Synthesizing Meta-Analyses

 Combining meta-analyses with different types of statistics is similar to synthesizing meta-analyses using the same type of statistics except for the appropriate conversion of summary statistics. We let ${\hat{\tau }}_{j}$ be an estimate for *τ* in the meta-analysis *j* using the standardized sample mean difference, ${\hat{\tau }}_{j^{\prime }}$ in the meta-analysis *j*′ using the log-transformed odds ratio, and ${\hat{\tau }}_{j^{\prime \prime }}$ in the meta-analysis *j*′′ using the correlation coefficient. These estimates take the similar form as (17) using data from the corresponding meta-analysis. The estimate for the summary effect in each meta-analysis is derived by using these estimates for *τ* and the variances of the effect estimates in the weights and is given by

(20) $$\begin{array}{c} {\hat{\theta }}_{j,m}=\frac{\sum_{i=1}^{k_{j}}{\tilde{w}}_{ij}^{*}{\hat{\theta }}_{ij,m}}{\sum_{i=1}^{k_{j}}{\tilde{w}}_{ij}^{*}},\\ {\hat{\theta }}_{j^{\prime },\text{OR}}=\frac{\sum_{i=1}^{k_{j^{\prime }}}{\tilde{w}}_{ij^{\prime }}^{*}{\hat{\theta }}_{ij^{\prime },\text{OR}}}{\sum_{i=1}^{k_{j^{\prime }}}{\tilde{w}}_{ij^{\prime }}^{*}},\\ {\hat{\theta }}_{j^{\prime \prime },z}=\frac{\sum_{i=1}^{k_{j^{\prime \prime }\;}}{\tilde{w}}_{ij^{\prime \prime }}^{*}{\hat{\theta }}_{ij^{\prime \prime },z}}{\sum_{i=1}^{k_{j^{\prime \prime }}}{\tilde{w}}_{ij^{\prime \prime }}^{*}}, \end{array}$$

where ${\tilde{w}}_{ij}^{*}=1/(\sigma_{ij}^{2}+{\hat{\tau }}_{j}^{2})$, ${\tilde{w}}_{ij^{\prime }}^{*}=1/(\sigma_{ij^{\prime }}^{2}+{\hat{\tau }}_{j^{\prime }}^{2})$, and ${\tilde{w}}_{ij^{\prime \prime }}^{*}=1/(\sigma_{ij^{\prime \prime }}^{2}+{\hat{\tau }}_{j^{\prime \prime }}^{2})$. To directly synthesize these meta-analysis results, we use a weighted average of converted effect estimates as follows:

(21) $$\begin{array}{c} \hat{\theta }=\frac{\sum_{j=1}^{J}w_{j}^{*}{\hat{\theta }}_{j,m}+\sum_{j^{\prime }=1}^{j^{\prime }}w_{j^{\prime }}^{*}{\hat{\theta }}_{j^{\prime },\text{OR}}/1.81+\sum_{j^{\prime \prime }=1}^{J^{\prime \prime }}2w_{j^{\prime \prime }}^{*}{\hat{\theta }}_{j,r}}{\sum_{j=1}^{J}w_{j}^{*}+\sum_{j^{\prime }=1}^{J^{\prime }}w_{j^{\prime }}^{*}+\sum_{j^{\prime \prime }=1}^{J^{\prime \prime }}w_{j^{\prime }}^{*}}, \end{array}$$

where ${\tilde{w}}_{j}^{*}=\sum_{j=1}^{J}{\tilde{w}}_{ij}^{*}$, ${\tilde{w}}_{j^{\prime }}^{*}=\sum_{j^{\prime }=1}^{J^{\prime }}{\tilde{w}}_{ij^{\prime }}^{*}$, and ${\tilde{w}}_{j^{\prime \prime }}^{*}=\sum_{j^{\prime \prime }=1}^{J^{\prime \prime }}{\tilde{w}}_{ij^{\prime }}^{*}$. The variance of  $\hat{\theta }$ is given by $\text{var}(\hat{\theta })=1/(\sum_{j=1}^{J}w_{j}^{*}+\sum_{j^{\prime }=1}^{J^{\prime }}w_{j^{\prime }}^{*}+\sum_{j^{\prime \prime }=1}^{J^{\prime \prime }}w_{j^{\prime }}^{*})$. The weights ${\tilde{w}}_{j}^{*}$, ${\tilde{w}}_{j^{\prime }}^{*}$, and ${\tilde{w}}_{j^{\prime \prime }}^{*}$ are usually calculated from the variances, *P* values, or the confidence intervals found in original meta-analyses being combined. Articles which only report effect estimates do not have sufficient information on the true effect and should be excluded in the synthesis. Following similar lines in previous sections, we can show that the estimated overall effect is approximately the same as the one by combining individual studies.

The effect estimates used in the expression (21) are already converted to the same type. The estimates reported in original meta-analyses may not be in the same form. For example, a meta-analysis using the correlation coefficient is likely to report the correlation coefficient instead of Fisher's *z*-score. Suppose ${\hat{\text{OR}}}_{j^{\prime }}$ is the estimated odds ratio in meta-analysis *j*′ and *r*_*j*′′_ is the estimated correlation coefficient in meta-analysis *j*′′. In terms of these indices and their variances originally reported in the meta-analyses, the overall effect estimate can be written as the following expression:

(22) θ^=(∑j=1Jwj,mθ^j,m+1.81∑j′=1J′wj′,ORln⁡(OR^j′)   +12∑j′′=1J′′wj′′,rln⁡(1+rj′′1−rj′′)) ×(∑j=1Jwj,m+1.812∑j′=1J′wj′,OR+14∑j′′=1J′′wj′′,r)−1,

where $w_{j,m}=1/\text{var}({\hat{\theta }}_{j,m})$, $w_{j^{\prime },\text{OR}}=1/\text{var}(\ln({\hat{\text{OR}}}_{j^{\prime }}))$, and *w*_*j*′′,*r*_ = 1/{var(*r*_*j*′′_)/(1−*r*_*j*′′^2^_)^2^}. And its variance is

(23) $$\begin{array}{c} \text{var}\left(\hat{\theta }\right)=\frac{1}{\sum_{j=1}^{J}w_{j,m}+1.81^{2}\sum_{j^{\prime }=1}^{J^{\prime }}w_{j^{\prime },\text{OR}}+1/4\sum_{j^{\prime \prime }=1}^{J^{\prime \prime }}w_{j^{\prime \prime },r}}. \end{array}$$

The proposed estimate in (22) is a summary standardized sample mean difference. Its expression represents two steps. The first step is to convert the odds ratios and correlation coefficients to the same scale of the standardized sample mean difference, and the second step is to calculate the overall mean difference from all meta-analyses. 

## 3. Examples

We illustrate the proposed methods in two examples. The first example illustrates that combining two meta-analyses using our methods produces the same overall effect size as conducting a meta-analysis from all includable primary studies based on either the fixed-effects model or the random-effects model. The second example illustrates the process of combining multiple meta-analyses of the same clinical question which have concordant findings. The calculation is conducted using the R package “metafor.”

The first example is based on a meta-analysis by Swift and Callahan [16], which compared treatment outcomes between appropriately and inappropriately matched groups using 26 individual studies [16]. The treatments in these studies include pharmacotherapy, cognitive behavioral therapy, and group therapy. The treatment outcomes range from substance use days to weight loss. All the studies used the Pearson correlation coefficient as the effect estimate, and the estimates and the confidence intervals are presented in Table 1. The summary correlation coefficient estimate using all 26 studies is *r* = 0.1334 with 95% confidence interval (0.0951,0.1717) based on a fixed-effects model. We conduct two separate meta-analyses based on studies 1–12 and studies 13–26 as cited in the reference section of the review in Swift and Callahan [16]. The summary effect sizes for these two meta-analyses are calculated to be ${\hat{\theta }}_{1}=0.1774$ with its sample variance being $\text{var}({\hat{\theta }}_{1})=0.00095$ and ${\hat{\theta }}_{2}=0.1041$ with its sample variance being $\text{var}({\hat{\theta }}_{2})=0.00064$. It is of interest to investigate whether combining these two effect sizes gives a similar result as conducting the meta-analysis using all 26 individual studies. We use the inverse of the sample variances as the weights and calculate the weighted average of the summary effect sizes. The resulting overall effect size is

(24) $$\begin{array}{c} {\hat{\theta }}_{f}=\frac{\left(\sum_{j=1}^{2}{\hat{\theta }}_{j}/\text{var}\left({\hat{\theta }}_{j}\right)\right)}{\left(\sum_{j=1}^{2}1/\text{var}\left({\hat{\theta }}_{j}\right)\right)}=0.1334, \end{array}$$

with its variance being $\text{var}({\hat{\theta }}_{f})=0.00038$. The 95% confidence interval is also (0.0951,0.1717). The results are identical to those found using all individual effect sizes indicating the potential utility of this procedure.

To further illustrate the technique we repeat the analyses with a random-effects model (REM). We again use the 26 studies from Swift and Callahan [16] and illustrate that the proposed summary effect size obtained under REM may differ slightly from the one achieved from combining all individual studies. Under the REM, the summary correlation coefficient using all 26 studies is $\hat{\theta }=0.1597$ with 95% confidence interval (0.0941, 0.2253). We repeat the process described above based on studies 1–12 and studies 13–26, respectively. Using the random-effects model, the summary correlation coefficients for these two meta-analyses are ${\hat{\tilde{\theta }}}_{1}^{*}=0.1827$ with its variance being $\text{var}({\hat{\tilde{\theta }}}_{1}^{*})=0.0027$ and ${\hat{\tilde{\theta }}}_{2}^{*}=0.1370$ with its variance being $\text{var}({\hat{\tilde{\theta }}}_{2}^{*})=0.0018$. Combining these two effect sizes produces an overall weighted mean correlated coefficient ${\hat{\theta }}_{r}=0.1553$, with its variance being $\text{var}({\hat{\theta }}_{r})=0.00108$. This variance is smaller than those of separate meta-analyses, indicating more accuracy in estimating the overall correlation coefficient by including more individual studies. The 95% confidence interval is (0.0909,0.2197). Although the estimates of the between-studies variance differ slightly across the two meta-analyses, combining the two pseudoanalyses produces a similar finding to that of combining all of the includable individual studies.

To illustrate the method for synthesizing different types of effect estimates, we create a synthetic dataset using the studies from Swift and Callahan [16]. We convert the correlation coefficients to standardized sample mean differences for studies 1–9 and to log-transformed odds ratios for studies 10–17. The estimates are kept unchanged for studies 18–26. The converted estimates and the corresponding confidence intervals are listed in Table 2. The variances of the estimates are obtained using (UL − LL)^2^/(2 × 1.96)^2^. We use the fixed-effects model to conduct meta-analyses on these three sets of studies. The summary effect estimates are ${\hat{\theta }}_{1,m}=0.4355$ with $\text{var}({\hat{\theta }}_{1,m})=0.0059$, ${\hat{\theta }}_{1,\text{OR}}=0.3304$ with $\text{var}({\hat{\theta }}_{1,\text{OR}})=0.0183$, and ${\hat{\theta }}_{1,r}=0.0711$ with $\text{var}({\hat{\theta }}_{1,r})=0.0011$. The overall effect estimate in mean difference is calculated using (22) to be $\hat{\theta }=0.2973$ with variance $\text{var}(\hat{\theta })=0.0017$. We see that this result is quite close to the summary effect size calculated from combining individual studies at the beginning of the section which is 0.2692 with the variance 0.0016 after converting to the mean effect size. This indicates that the proposed procedure for directly combining results from meta-analyses produces similar results as the ideal but much more tedious procedure which instead combines individual studies subtracted from meta-analyses.

 The anterior cruciate ligament (ACL) is a commonly reconstructed ligament of the knee. The meta-analyses conducted by Biau et al. [17] and M. C. Forster and I. W. Forster [18] indicate that the graft failure using hamstring autografts is not significantly different compared with bone-patellar tendon-bone autografts. We sought to apply our approach to synthesize these two meta-analyses on the graft choices in the ACL reconstruction. The summary odds ratios of graft fracture in Biau et al. [17] are ${\hat{\text{OR}}}_{1}=1.33$ with the 95% confidence interval (0.73,2.44) and ${\hat{\text{OR}}}_{2}=1.09$ with the 95% confidence interval (0.40,2.96) in M. C. Forster and I. W. Forster [18]. We first convert the odds ratios and their confidence limits to the natural log scale since meta-analyses using odds ratios are commonly conducted using the weighted average of the log-transformed odds ratios. After the conversion, we obtain the log-transformed odds ratios ${\hat{\theta }}_{1,\text{OR}}=0.2852$ with the variance 0.9048 and ${\hat{\theta }}_{2,\text{OR}}=0.0862$ with the variance 0.3607. The resulting overall estimate of log odds ratio is

(25) $$\begin{array}{c} \hat{\theta }=\overset{2}{\underset{j=1}{\sum }}\left(\frac{{\hat{\theta }}_{j,\text{OR}}/\text{var}\left({\hat{\theta }}_{j,\text{OR}}\right)}{\sum_{j=1}^{2}\left(1/\text{var}\left({\hat{\theta }}_{j,\text{OR}}\right)\right)}\right)=0.2321, \end{array}$$

with the variance 0.0695. This variance is smaller than either of the meta-analyses due to the inclusion of more studies. The 95% confidence interval of $\;\;\hat{\theta }\;\;$ is (− 0.2846, 0.2321). Converting back to the original scale, the overall estimated odds ratio based on the two meta-analyses is 1.2613 with 95% confidence interval (0.7523, 1.2613). Although this confidence interval does not show significant difference between two graft choices, it is narrower than those in Biau et al. [17] and M. C. Forster and I. W. Forster [18]. This is expected since the overall estimated odds ratio calculated across meta-analyses provides a more accurate indicator than those calculated from separate meta-analyses.

## 4. Discussion

Despite the advantages of meta-analyses over traditional narrative review methods, these techniques are not without limitations. As is the case with primary studies, multiple meta-analyses often produce conflicting findings even when they examine the same body of literature [7] given the numerous methodological decisions that are made during the review process. These decisions affect the search strategy, coding of studies, moderators examined, inclusion/exclusion criteria, and the reporting of effect sizes, and each has the potential to impact the review findings. Despite the transparency of the method, there is always a certain degree of subjectivity in research synthesis [14]. We have presented methods which can synthesize meta-analysis findings with minimal subjectivity.

Attempting to make statements about the overall findings of a body of systematic review literature raises a number of challenges. Part of the challenge is the lack of consistency in how authors report the findings of meta-analyses [19, 20]. Since no standardized reporting procedures have been adopted, there is a great deal of variability in how meta-analytic findings are reported both within and across academic disciplines. Additionally, many extant meta-analyses do not completely report effect sizes; confidence intervals, variance measures, and precise *P* values are often omitted. This lack of consistency and quality assurance is a major limitation of this body of research and potentially limits the transportability of meta-analysis findings [21]. Like primary studies, meta-analyses on similar constructs may differ from one another in many ways. To address this issue and make meta-analyses more compatible and therefore more appropriate for synthesis and better equipped to inform practice, it is apparent that the research community needs to continue to work towards adopting a set of standardized best practices for conducting and reporting meta-analyses in behavioral health (see, e.g., [22]).

While the examples provided here illustrate that it is possible to calculate summary effect sizes across multiple meta-analyses of the same intervention, our testing of these techniques is still ongoing. A potential barrier to this approach is the lack of independence that results when multiple meta-analyses include many of the same primary studies. Treating these different outcomes as independent can produce incorrect estimates of the variance for the summary effect, but it does not invalidate the calculation of the effect size itself. This can be seen from (4) for combining individual studies. For example, suppose *J* meta-analyses use the exactly same set of studies. Equation (4) becomes

(26) $$\begin{array}{c} \hat{\theta }=\frac{J\sum_{i=1}^{k_{1}}w_{i1}^{*}{\hat{\theta }}_{i1}}{J\sum_{i=1}^{k_{1}}w_{i1}^{*}}, \end{array}$$

which gives the same estimate as the summary effect estimate in either of the meta-analyses. Ideally, the resulting variance of $\hat{\theta }$ should be the same as the one in either of the meta-analyses. But instead, we have

(27) $$\begin{array}{c} \text{var}\left(\hat{\theta }\right)=\frac{1}{J\sum_{i=1}^{k_{1}}w_{i1}^{*}}, \end{array}$$

which tends to be considerably smaller than the appropriate value of 1/∑_*i*=1_^*k*_1_^ *w*_*i*1_*. This limitation makes the proposed method more appropriate for meta-analyses with no or a limited number of overlapping studies. A possible inflation factor of *J* can be multiplied with the variance in this case to correct for the biased variance estimator. More generally, with *p* overlapping studies in all meta-analyses, the unbiased variance of the summary estimator can be written as

(28) var(θ^)=1∑j=1J∑i=1kwij   ×J2∑i=1pwi1+∑j=1J∑i=p+1kwijJ∑i=1pwi1+∑j=1J∑i=p+1kwij=1∑j=1J∑i=1kwij×IF,

where the inflation factor IF corrects for the underestimated variance and we have IF ≥ 1. If *w*_*ij*_'s can be retrieved from the overlapped studies, the factor can be precisely calculated. Otherwise, the investigator may have to guess an appropriate value for the inflation factor.

While further research is needed to refine the technique proposed in this paper, the findings of these initial validations suggest the utility of this method for combining mean effect sizes from multiple meta-analyses of the same intervention or treatment. The methodology detailed in this paper has several possible applications. The technique has its most utility when several meta-analyses have been conducted on the same treatment and have produced varying results. Even when multiple meta-analyses report similar findings regarding the magnitude and direction of effect sizes, the proposed technique can be used to summarize across the extant meta-analytic evidence base. The technique also offers a solution when meta-analyses have been conducted to update prior research syntheses; rather than ignoring findings predating the inclusion criteria of the updated study, this technique can be used to summarize the full range of available evidence. These and other potential applications make this a particularly appealing technique for researchers and practitioners alike who are faced with the challenge of summarizing the rapidly expanding body of meta-analytic research in medicine and many other scientific disciplines.

## Figures and Tables

**Table 1:** Effect estimates with lower limits (LL) and upper limits (UL) of 95% confidence interval (CI).

Study number	*r *	95% CI		var(*r*)
		LL	UL	
1	0.25	0.04	0.44	0.0104
2	0.21	−0.02	0.43	0.0132
3	0.18	0.00	0.35	0.0080
4	0.05	−0.15	0.24	0.0099
5	0.17	0.00	0.33	0.0071
6	0.33	0.09	0.53	0.0126
7	0.26	0.02	0.47	0.0132
8	0.51	0.24	0.71	0.0144
9	0.25	0.03	0.45	0.0115
10	−0.26	−0.45	−0.04	0.0109
11	0.23	−0.04	0.48	0.0176
12	0.04	−0.24	0.32	0.0204
13	0.50	0.25	0.69	0.0126
14	0.11	−0.28	0.47	0.0366
15	0.15	−0.04	0.33	0.0089
16	0.10	0.00	0.21	0.0029
17	0.04	−0.19	0.27	0.0138
18	0.04	−0.12	0.21	0.0071
19	0.12	−0.03	0.27	0.0059
20	0.55	0.15	0.80	0.0275
21	−0.07	−0.31	0.19	0.0163
22	0.18	−0.26	0.56	0.0438
23	0.23	0.00	0.44	0.0126
24	0.11	−0.14	0.34	0.0150
25	0.21	−0.14	0.52	0.0283
26	−0.04	−0.15	0.07	0.0031

**Table 2:** Effect estimates of different types with lower limits (LL) and upper limits (UL) of 95% confidence interval (CI). Summary effect estimates are calculated based on a fixed-effects model for three sets of studies.

Study no.	${\hat{\theta }}_{m}$	95% CI		Study no.	${\hat{\theta }}_{\text{OR}}$	95% CI		Study no.	${\hat{\theta }}_{r}$	95% CI	
		LL	UL			LL	UL			LL	UL
1	0.52	0.08	0.98	10	−0.97	−1.82	−0.14	18	0.04	−0.12	0.21
2	0.43	−0.04	0.95	11	0.86	−0.14	1.98	19	0.12	−0.03	0.27
3	0.37	0.00	0.75	12	0.14	−0.89	1.22	20	0.55	0.15	0.80
4	0.10	−0.30	0.49	13	2.09	0.93	3.45	21	−0.07	−0.31	0.19
5	0.35	0.00	0.70	14	0.40	−1.06	1.93	22	0.18	−0.26	0.56
6	0.70	0.18	1.25	15	0.55	−0.14	1.27	23	0.23	0.00	0.44
7	0.54	0.04	1.06	16	0.36	0.00	0.78	24	0.11	−0.14	0.34
8	1.19	0.49	2.02	17	0.14	−0.70	1.02	25	0.21	−0.14	0.52
9	0.52	0.06	1.01					26	−0.04	−0.15	0.07
